# vcf2fhir: a utility to convert VCF files into HL7 FHIR format for genomics-EHR integration

**DOI:** 10.1186/s12859-021-04039-1

**Published:** 2021-03-02

**Authors:** Robert H. Dolin, Shaileshbhai R. Gothi, Aziz Boxwala, Bret S. E. Heale, Ammar Husami, James Jones, Himanshu Khangar, Shubham Londhe, Frank Naeymi-Rad, Soujanya Rao, Barbara Rapchak, James Shalaby, Varun Suraj, Ning Xie, Srikar Chamala, Gil Alterovitz

**Affiliations:** 1 Elimu Informatics, 1160 Brickyard Cove Rd Ste 200, Richmond, CA 94801-4173 USA; 2 Department of Pathology, Immunology and Laboratory Medicine, University of Florida, Gainesville, FL USA; 3 Intermountain Healthcare, Salt Lake City, UT USA; 4 Division of Human Genetics, Cincinnati Children’s Hospital Medical Center and Department of Pediatrics, University of Cincinnati College of Medicine, Cincinnati, OH USA; 5 Computational Health Informatics Program, Boston Children’s Hospital, Boston, MA USA; 6 Leap of Faith, Libertyville, IL USA; 7 Lexington High School, Lexington, MA USA; 8 Biomedical Cybernetics Laboratory, Department of Medicine, Brigham and Women’s Hospital, Boston, MA USA; 9 Brigham and Women’s Hospital, Boston, MA USA; 10 Harvard/MIT Division of Health Sciences and Technology, Harvard Medical School, Boston, MA USA

**Keywords:** FHIR, Clinical genomics, SMART-on-FHIR, EHR integration, Next-generation sequencing

## Abstract

**Background:**

VCF formatted files are the lingua franca of next-generation sequencing, whereas HL7 FHIR is emerging as a standard language for electronic health record interoperability. A growing number of FHIR-based clinical genomics applications are emerging. Here, we describe an open source utility for converting variants from VCF format into HL7 FHIR format.

**Results:**

vcf2fhir converts VCF variants into a FHIR Genomics Diagnostic Report. Conversion translates each VCF row into a corresponding FHIR-formatted variant in the generated report. In scope are simple variants (SNVs, MNVs, Indels), along with zygosity and phase relationships, for autosomes, sex chromosomes, and mitochondrial DNA. Input parameters include VCF file and genome build (‘GRCh37’ or ‘GRCh38’); and optionally a conversion region that indicates the region(s) to convert, a studied region that lists genomic regions studied by the lab, and a non-callable region that lists studied regions deemed uncallable by the lab. Conversion can be limited to a subset of VCF by supplying genomic coordinates of the conversion region(s). If studied and non-callable regions are also supplied, the output FHIR report will include ‘region-studied’ observations that detail which portions of the conversion region were studied, and of those studied regions, which portions were deemed uncallable. We illustrate the vcf2fhir utility via two case studies. The first, 'SMART Cancer Navigator', is a web application that offers clinical decision support by linking patient EHR information to cancerous gene variants. The second, 'Precision Genomics Integration Platform', intersects a patient's FHIR-formatted clinical and genomic data with knowledge bases in order to provide on-demand delivery of contextually relevant genomic findings and recommendations to the EHR.

**Conclusions:**

Experience to date shows that the vcf2fhir utility can be effectively woven into clinically useful genomic-EHR integration pipelines. Additional testing will be a critical step towards the clinical validation of this utility, enabling it to be integrated in a variety of real world data flow scenarios. For now, we propose the use of this utility primarily to accelerate FHIR Genomics understanding and to facilitate experimentation with further integration of genomics data into the EHR.

## Background

Variant Call Format (VCF) formatted files [1] are the lingua franca of next-generation sequencing whereas the HL7 FHIR specification [2] is emerging as a standard language for electronic health record (EHR) interoperability. FHIR represents a novel approach to interoperability, being comprised of atomic 'resources' that can be assembled in various ways to meet specific use cases. The HL7 community has defined FHIR resources for lab observations, for medical conditions, for medications, for allergies, for vital signs, and much more. GA4GH has announced plans for an implementation of the Phenopackets specification in FHIR [3]. Recently, HL7 published the FHIR Genomics Implementation Guide (aka 'FHIR Genomics') [4] that defines a FHIR-based representation of variants, haplotypes, genotypes, variant annotations, and more; and the FHIR minimal Common Oncology Data Elements guide (aka 'FHIR mCode') [5] that defines a core set of structured data elements, including genomic data elements, for oncology. Large research projects such as eMERGE [6] and CSER [7] are exploring the use of FHIR Genomics, and evidence shows the clinical informatics community, including the clinical genomics informatics community, moving towards greater and greater use of FHIR-based solutions [8–13].

But while there is tremendous momentum driving the adoption of FHIR in EHRs, it represents a novel technology to clinical laboratories, most of whom already use the HL7 Version 2 interoperability specifications; and next-generation sequencing (NGS) centers, most of whom are intimately familiar with the VCF format. On top of this, FHIR, being a relatively new standard, presents challenges related to its maturity [14].

Here, we provide a vcf2fhir translation utility that when fed a VCF, will convert it into a FHIR Genomics report. Prior conversion utilities based on earlier versions of FHIR have been since deprecated [15], and we are not aware of other utilities providing this function. We propose that this utility can facilitate the migration to FHIR Genomics, and we anticipate that the tool will ultimately be used in a variety of EHR data integration pipelines.

### Implementation

**Fig. 1:**
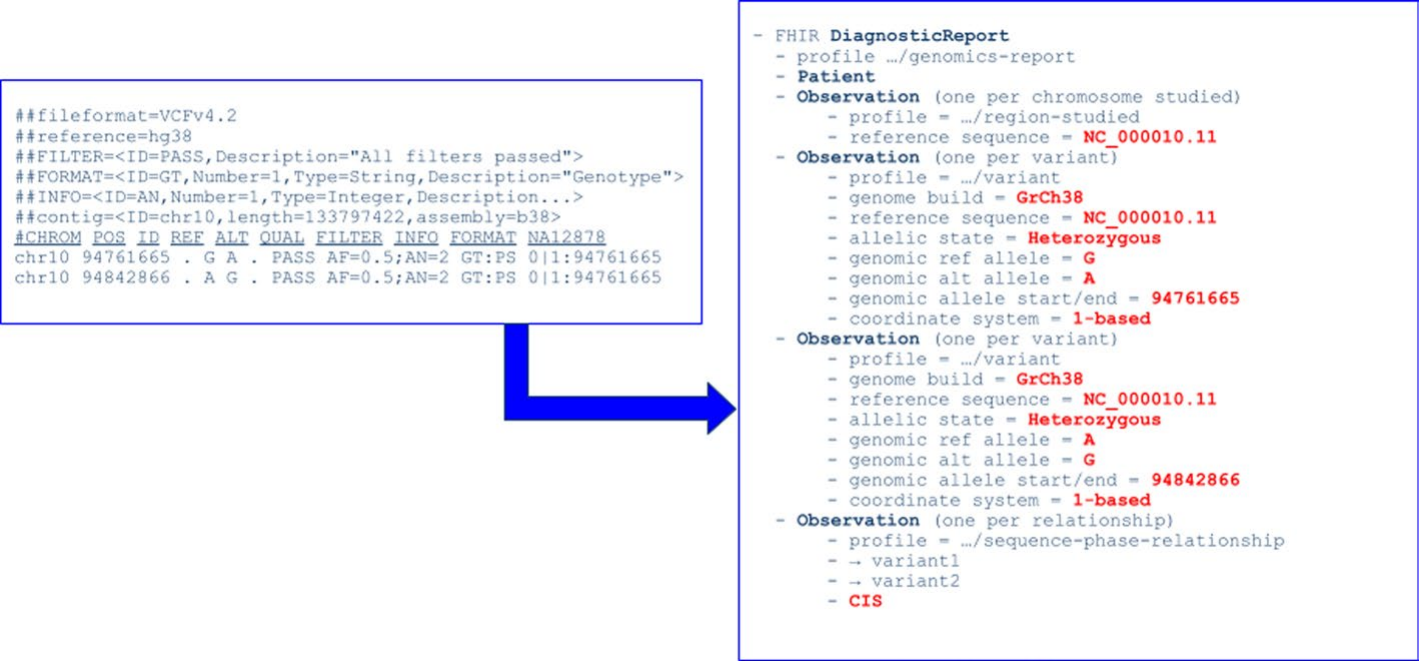
vcf2fhir conversion. The vcf2fhir utility takes a VCF file as input and outputs a FHIR Genomics report in JSON format. A simplified conceptual representation of the FHIR report is shown here to illustrate the main components. (VCF is a text file. It contains meta-information lines (prefixed with ‘##’), a header line (prefixed with ‘#’), and tab-delimited data lines each containing information about a variant. The CHROM and POS fields indicate the location of the variant. REF indicates the reference allele while ALT indicates the alternate allele. FILTER indicates if the variant call has passed applied filters. INFO is a semicolon-separated series of fields that further characterize a variant. FORMAT is colon-separated list of fields that characterize the genotype. Fields defined in FORMAT are valued for each tested sample, such as the NA12878 sample shown. A FHIR Genomics report is represented as a FHIR Diagnostic Report that contains information about the patient, and a set of observations. Each observation in the FHIR Genomics report conforms to a defined FHIR Observation ‘profile’ that constrains the information conveyed. In particular, the report includes zero or more ‘region-studied’ observations, ‘variant’ observations, and ‘sequence-phase-relationship’ observations, which are described in the text)

Conceptually, the vcf2fhir utility takes a VCF as input and outputs a FHIR Genomics report, as shown in Fig. 1. We currently convert simple variants (SNVs, MNVs, Indels), along with zygosity and phase relationships, for autosomes, sex chromosomes, and mitochondrial DNA. Input parameters include VCF file (text-based or bgzipped) and genome build ('GRCh37’ or 'GRCh38’); and optionally a conversion region that indicates the region(s) to convert, a studied region that lists genomic regions studied by the lab (generally defined based on the specific test protocol used by a lab to generate the sequencing data), and a non-callable region that lists studied regions deemed uncallable by the lab (e.g. due to low depth of sequencing coverage, enrichment specific target issues and other platform specific reasons). Output is a FHIR Genomics Diagnostic Report [4] (in JSON format) that contains converted variants.

Conversion can be limited to a subset of VCF by supplying conversion region(s). If studied and non-callable regions are also supplied, the output FHIR report will include 'region-studied' observations that detail which portions of the conversion region were studied, and of those studied regions, which portions were deemed uncallable.

**Fig. 2:**
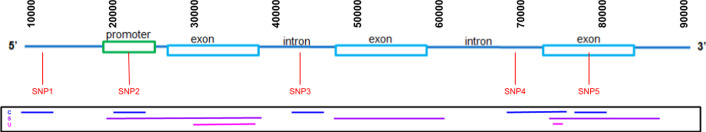
vcf2fhir conversion showing region-studied capabilities. See text for details. *SNP* single nucleotide polymorphism, *C* conversion region (blue lines), *S* studied region (purple lines), *U* uncallable region (magenta lines)

We illustrate the region-studied capabilities in Fig. 2. In this scenario, whole exome sequencing has been used to generate a VCF file. A user is interested in a patient's genotype at five loci, labeled SNP1-SNP5. A conversion region, shown in blue, is supplied, directing the software to convert positions 10,000–12,000, 20,000–22,000, 41,000–43,000, 68,000–73,000, 75,000–80,000. A studied region, shown in purple, is supplied, showing that positions 18,000–37,000, 46,000–58,000, 71,000–83,000 were studied. An uncallable region, shown in magenta, is supplied, showing that positions 30,000–36,000, 71,500–72,000 were deemed uncallable by the producing laboratory. Software will convert all variants in the conversion region. In addition, the generated FHIR report will include a region studied observation showing that of the requested conversion regions, ranges studied include 20,000–22,000, 71,000–73,000, 75,000–80,000; and uncallable region includes 71,500–72,000. As a result, the user can determine, for instance, that the absence of a variant call at SNP1 locus is because the region was not studied, whereas the absence of a variant call at SNP5 locus implies homozygous reference genotype at that position.

Conversion translates each VCF row into a corresponding FHIR-formatted variant that conforms to the FHIR 'variant' profile (aka 'FHIR Variant') [16]. Mapping from VCF to FHIR Variant is straightforward where there is a one to one correspondence between fields (e.g. both VCF and FHIR Variant have a field for reference allele, alternate allele, variant position). Where data models differ, more complex mapping rules are needed—for instance, VCF's genotype information (e.g. FORMAT.GT = 0/1) is translated into FHIR Variant's allelic state (e.g. heterozygous). In the uncommon case where a VCF row indicates a compound heterozygous genotype (e.g. FORMAT.GT = 1/2), we create two heterozygous FHIR variants. Where the VCF indicates absence of variant (FORMAT.GT = 0/0), we set alternate allele to equal the reference allele in FHIR.

Within a VCF file, variants may or may not have been normalized by one of several algorithms (such as [17]). Within FHIR, variants can be represented in several formats (VCF-like format, HGVS-like format, etc.). vcf2fhir conversion serves as 'syntactic normalization', in that all variants in the generated FHIR report are represented using a consistent set of FHIR fields. vcf2fhir does not perform 'semantic normalization', but rather, mirrors the VCF record in FHIR (e.g. same values for reference allele, alternate allele, variant position).

Conversion creates FHIR phase relationships where two heterozygous variants are asserted to be in a cis (both variants on the same chromosome) or trans (each variant is on a different chromosome) configuration in the VCF file. In this case, vcf2fhir creates a sequence phase relationship observation with a relationship of 'Cis' or 'Trans', as shown in Table 1.

**Table 1:** Relationship between VCF and FHIR phase relationships

VCF rows	FHIR phase relationship
6 18142205. C T... GT:PS 1|0:18142205 6 18142422. A C... GT:PS 0|1:18142205	Heterozygous variants are TRANS
6 18142289. A G... GT:PS 1|0:18142289 6 18142308. A G... GT:PS 1|0:18142289	Heterozygous variants are CIS

Phase relationships in VCF represented using the FORMAT.PS field are converted into FHIR 'CIS' and 'TRANS' relationships

Sex chromosome conversion translates chrX and chrY calls as they exist in the VCF. Many VCF calling pipelines mask the pseudoautosomal regions (PAR) of chrY, as described by 1000 Genomes project [18]. As a result, we commonly see diploid calls in PAR chrX and absence of calls in PAR chrY for males, with haploid calls in non-PAR regions of chrX and chrY. In females, we commonly see only diploid calls for chrX, and no calls for chrY. Where a VCF row indicates a haploid call (i.e. FORMAT.GT has 1 allele), we translate to a FHIR variant having an allelic state of hemizygous.

Mitochondrial DNA conversion assumes haploid calls, as described by the VCF specification [1]. FHIR Variant's allelic state is based on allelic depth (FORMAT.AD) divided by read depth (FORMAT.DP). If FORMAT.AD/FORMAT.DP is greater than 99% then allelic state is set to homoplasmic, and is otherwise set to heteroplasmic.

Conversion examples and a more detailed description of the conversion process are available through the github site.

## Results and discussion

While all sequencing labs are intimately familiar with VCF, many are far less familiar with FHIR, despite its growing EHR adoption. Potential uses for this utility include helping a sequencing lab understand how to generate a FHIR Genomics report, helping an EHR or clinical decision support (CDS) application understand how to compute on structured genomics data in FHIR Genomics format, stream-lining the development of clinical FHIR-based applications, and helping the bioinformatics community gain familiarity with FHIR. The utility is also finding use as a component of larger systems, as described in the following case studies. We anticipate that the tool will ultimately be used in a variety of sequencing lab to EHR data integration pipelines.

The vcf2fhir utility has been tested in several HL7 FHIR Connectathons [19, 20], which are collaborative hands-on FHIR integration testing events held tri-annually; as part of a pharmacogenomics (PGx) CDS pipeline [9]; and via the case studies described below. Planned future software development includes enhancing the conversion logic to accommodate VCF rows representing structural variants (i.e. rows that contain an INFO.SVTYPE field).

### Implementation case study: SMART Cancer Navigator

The SMART Cancer Navigator [21] is a web application that offers CDS by linking patient EHR information in the FHIR format to cancerous gene variants. It queries multiple genetic databases to get gene variant information, which it displays in an organized fashion so that clinicians and patients can view information regarding gene variants, potential diseases resulting from the variant, and possible treatments. The app accesses patient data from FHIR servers, such as Veterans Affairs and Center for Medicare and Medicaid Services, to obtain information about patient demographics and medical conditions. The Navigator uses the vcf2fhir converter to get information regarding a patient's gene variants and display them in the application.

**Fig. 3:**
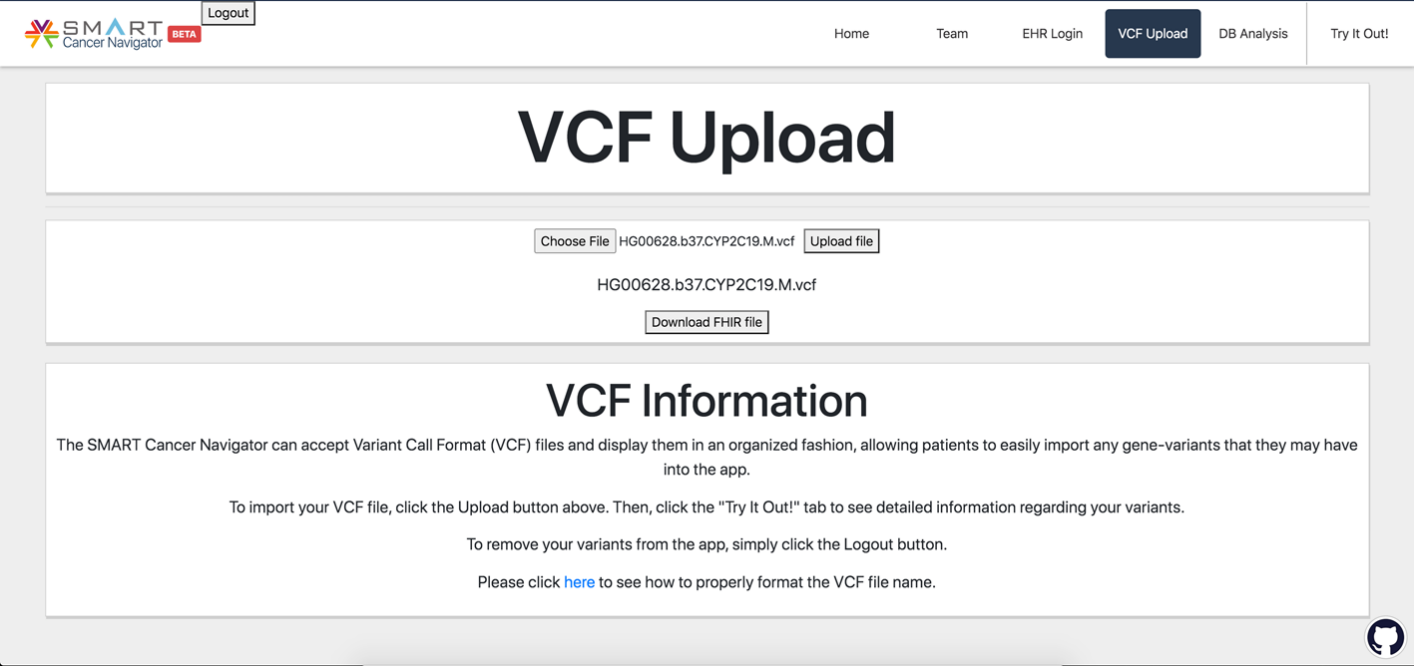
Loading a VCF file into the SMART Cancer Navigator. To upload a file, the user clicks the “Choose File” button, selects a VCF file from their computer, and then clicks the “Upload file” button. Once a VCF file is uploaded, the user can click the “Download FHIR file” button to have their FHIR file downloaded into their computer’s “Downloads” folder. Error messages show when a user has not submitted a file and when the VCF file is invalid

Conversion is initiated by uploading a valid VCF file to the Navigator (Fig. 3). Because of legacy constraints, we currently require that the VCF file be limited to variants for a single gene, and that the gene name be included in the VCF file name. Once uploaded, the VCF file is sent by the Cancer Navigator to the converter over an HTTP POST request. We do not include a coverage region or uncallable region in the request because we want the entire VCF file converted into FHIR format. The server receiving the request hosts the vcf2fhir converter, and converts the VCF file into FHIR Genomics format for return to the client in the form of a file. The use of Cross-Origin Resource Sharing allows for applications with different domains to send HTTP calls to the server application [22].

Upon receipt of the FHIR Genomics file, the Navigator extracts each variant into a specified array. Each array index corresponds to a variant. Information such as the chromosome position and the reference and alternate alleles are stored here, and are labeled by LOINC codes [23]. Next, the Navigator queries MyVariant.info for a list of known variants for the gene in question. From this query, the hgvs ID of the variant, the variant name, and the entrezID of the gene are obtained, along with the chromosome position and reference and alternate alleles. The Navigator compares patient variants against the list obtained from MyVariant.info, storing any that are in common. The Navigator then gets detailed information about each of the variants in common via calls to MyVariant.info and MyGene.info [24, 25]. The MyVariant.info call uses the hgvs ID that was collected in the first query, and gets more information from the knowledge base, such as a description of the variant, the type of mutation that caused the variant, and whether the variant is somatic. The MyGene.info call uses the previously collected entrez ID, and gets information such as a description of the gene, whether the gene is protein-coding, and its position on the chromosome.

**Fig. 4:**
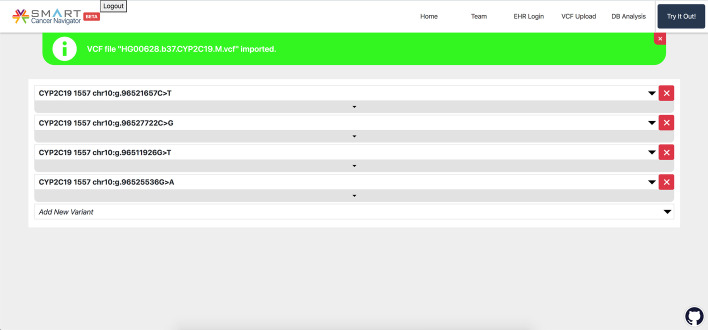
SMART Cancer Navigator variant viewer. The main page of the application after a VCF file has been uploaded. The page populates with variants that were found in common between the converted FHIR file and the gene-variant knowledge base queries

When the user navigates to the main page of the SMART Cancer Navigator after uploading their VCF file, they will see a page pre-populated with variants, based on the variants that were found in the FHIR file generated by the vcf2fhir converter (Fig. 4). In addition, the Navigator gives the user the option to download their FHIR Genomics file (Fig. 3).

### Implementation case study: Precision Genomics Integration Platform

NGS can identify thousands to millions of variants, whose clinical significance can change over time as our knowledge evolves. To manage such a large volume of (dynamic) results, many institutions are exploring the storage of genomic data outside the EHR, in a genomic data server, also referred to as a Genomic Archiving and Communication System (GACS) [26, 27]. A GACS stores sequence data generated from a sequencing laboratory and is analogous in many ways to a Picture Archiving and Communication System (PACS), which stores image files that are not suitable to store directly in an EHR. This trend has led the US Office of the National Coordinator’s (ONC) Sync for Genes project to emphasize the need for pilots that test the use of FHIR for GACS integration with EHRs [28]. In support of this effort, HL7 has developed a 'find-subject-variants' operation [29] that returns a set of FHIR-formatted variants within a specified range from GACS.

**Fig. 5:**
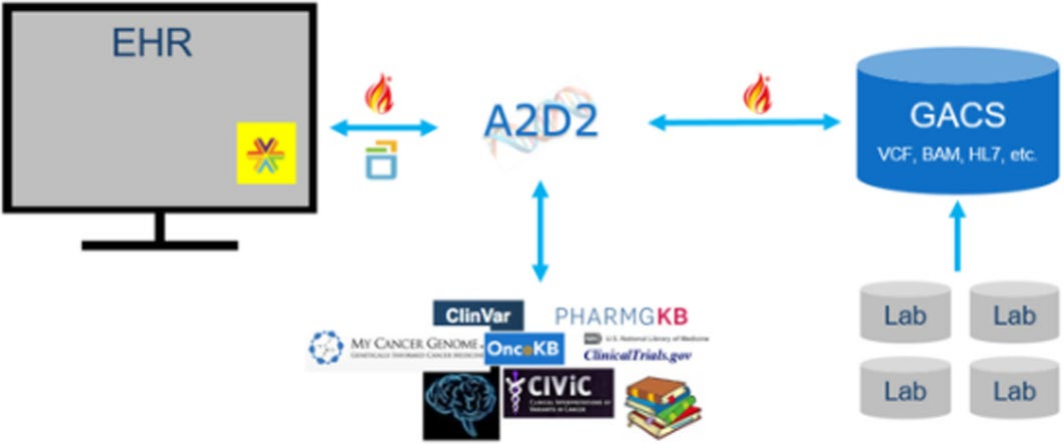
Precision genomics integration platform. Platform components include a FHIR-enabled genomic data server (GACS) and a workflow/CDS engine (A2D2). A2D2 can computationally intersect a patient's FHIR-formatted clinical and genomic data with knowledge bases in order to provide on-demand delivery of contextually relevant genomic findings and recommendations to the EHR

The Precision Genomics Integration Platform, shown in Fig. 5, is a soon-to-be-released open source platform that includes an implementation of the HL7 Genomics find-subject-variants operation, based on the vcf2fhir translator. The platform provides access to a patient's FHIR-formatted clinical and genetic data along with capabilities for the on-demand delivery of contextually relevant genomic findings and recommendations, via multiple EHR integration points. Primary components of the platform include a FHIR-enabled GACS and a central CDS and workflow engine known as 'A2D2’. GACS is a scalable cloud-hosted primary repository of genomic data. It can store raw bioinformatics files (e.g. VCF, BAM) and genomics data received in other formats (e.g. HL7 V2, FHIR).

Where GACS is storing VCF files, it uses the vcf2fhir converter to dynamically translate VCF variants in the requested range into FHIR format to be returned to the caller—which in the case of this platform is A2D2. It should be noted that vcf2fhir is designed as a stand-alone utility that can be invoked as part of a pipeline. As such, input parameters include the VCF file itself, along with conversion, studied, and uncallable regions. On the other hand, the HL7 find-subject-variants operation is designed as a query against a genomic data server that already houses genomic data and its associated metadata. As such, the find-subject-variants operation input parameters only include a patient ID and a conversion region. We have extended the operation with an additional parameter that allows the client to indicate when they want uncallable regions included in the returned FHIR Genomics file.

Operationally, GACS receives an HTTP GET request from A2D2 which includes a patient ID and a conversion region (a reference sequence identifier and an integer range), and optionally a flag to include uncallable regions. GACS uses the conversion parameters to extract the relevant region from the patient’s VCF, which is then handed to the converter, where the entire extracted slice is converted into FHIR Genomics format. The FHIR report includes the region-studied observation in all cases, and in addition, includes uncallable subregions when specifically requested.

**Fig. 6:**
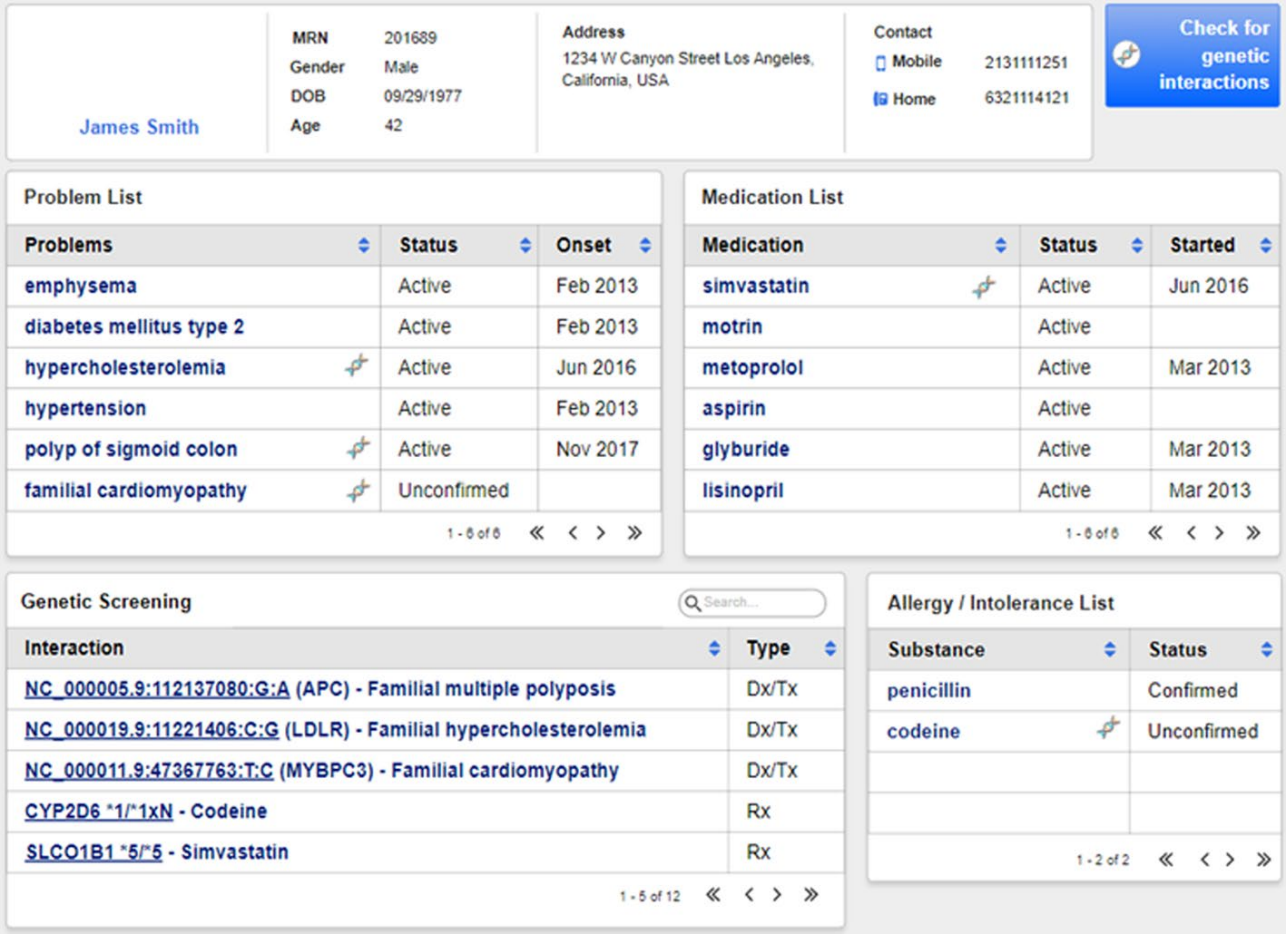
Face sheet application with genomic annotations. SMART-on-FHIR application that surfaces identified genomic interactions. (Shown is a fully synthetic patient)

The resulting FHIR Genomics report is returned to A2D2, the primary orchestrator of the Platform. External knowledge (e.g. ClinVar variants [30], CPIC [31] and PharmGKB [32] PGx rules) can be integrated into A2D2 via APIs or as rules encoded in Drools, and together with obtained clinical data, can be compared against a patient’s variants in the determination of various genomic interactions. Leveraging these components, we have developed a PGx order-entry CDS service based on the CDS Hooks protocol [33] integrated with Cerner’s EHR [9] and a SMART-on-FHIR Face Sheet application shown in Fig. 6 that computes and displays a wide range of identified genetic annotations. A more detailed description of the open source platform will be the subject of a future manuscript.

### Effectiveness and limitations

The case studies described above, along with experience in HL7 Connectathons and prior PGx CDS integration, illustrate the potential role of the vcf2fhir utility in enabling novel approaches to genomics-EHR integration. A common method today of integrating genomic results into EHRs is as verbose PDF reports received from the laboratory. These textual reports are neither ideal for clinicians nor for CDS—they contain only a slice of key variants and a point-in-time snapshot of interpretations; they are difficult and time-consuming to review; clinicians do not remember interactions mentioned in the reports when making relevant decisions, and they do not provide structured data needed for CDS. While EHR vendors are enhancing their products in anticipation of structured genomic findings, it is likely that such solutions will be incomplete—NGS can identify thousands to millions of variants, whose clinical significance can change over time as our knowledge evolves. Today's EHRs are not designed to manage such a large volume of (dynamic) results. On the other hand, housing genomic data in a separate genomic data server, wrapped by a set of FHIR APIs, in communication with the EHR and/or an intervening CDS engine, offers exciting possibilities for managing a person’s entire genome, managing evolution in our understanding of a person’s genome, and for provision of contextually relevant genomics findings and recommendations at the point of care.

That said, vcf2fhir has known limitations. An up to date list of known issues is available on the github site. These issues range from simple bug fixes (e.g. graceful handling of an unknown chromosome) to functional enhancements (e.g. support for structural variant conversion). Carefully understood, these limitations can be accommodated (e.g. don’t use the utility if your scenario includes structural variants). But even as these limitations are resolved, there will likely be additional GACS-based scenarios that require the creation of different FHIR APIs. For instance, vcf2fhir is not ideally suited for computing polygenic risk scores where one must look at the genotype of thousands of SNPs across a person’s genome. It is likely that a range of patient- and population-level genotype and phenotype operations will be necessary—a task which the HL7 Clinical Genomics Committee has recently embarked upon.

## Conclusions

Experience to date shows that the vcf2fhir utility can be effectively woven into clinically useful genomic-EHR integration pipelines. Additional testing will be a critical step towards the clinical validation of this utility, enabling it to be used in a variety of sequencing lab to EHR data flow scenarios. For now, we propose the use of this utility primarily to accelerate FHIR Genomics understanding and to facilitate experimentation with further integration of genomics data into the EHR.

## Availability and requirements

- **Project name**: vcf2fhir
- **Project home page**: https://github.com/elimuinformatics/vcf2fhir
- **Operating system(s)**: Platform independent
- **Programming language**: Python
- **License**: Apache 2.0
- **Any restrictions to use by non-academics**: None

## Data Availability

Not applicable.

## Abbreviations

BAM: Binary sequence alignment file
CDS: Clinical decision support
CPIC: Clinical Pharmacogenetics Implementation Consortium
CSER: Clinical Sequencing Evidence-Generating Research consortium
DNA: Deoxyribonucleic acid
EHR: Electronic health record
eMERGE: Electronic Medical Records and Genomics Network
FHIR: Fast Healthcare Interoperability Resources
GA4GH: Global Alliance for Genomics and Health
GACS: Genomic Archiving and Communication System
HL7: Health Level Seven Standards Organization
HGVS: Human Genome Variation Society
Indel: Insertion or deletion
NGS: Next-generation sequencing
ONC: United States Office of the National Coordinator for Health Information Technology
PACS: Picture Archiving and Communication System
PAR: Pseudoautosomal region
PGx: Pharmacogenomics
SNP: Single nucleotide polymorphism
SNV: Single nucleotide variant
VCF: Variant call format
